# Influence of meteorological and cosmic conditions on dose rate in the environment of the Polish Polar station Hornsund

**DOI:** 10.1007/s00484-025-02999-0

**Published:** 2025-08-13

**Authors:** Zenon Nieckarz, Marek Kubicki

**Affiliations:** 1 Marian Smoluchowski Institute of Physics, Jagiellonian University, ul. prof. Stanisława Łojasiewicza 11, Kraków, 30-348 Poland; 2 Institute of Geophysics, Polish Academy of Sciences, ul. Księcia Janusza 64, Warszawa, 01-452 Poland

**Keywords:** Ionization of air, Dose rate, Cosmic radiation, Solar activity, Polar region

## Abstract

This study presents, for the first time, the results of long-term measurements of radiation dose rates from cosmic and terrestrial gamma rays conducted at the Polish Polar Station Hornsund (southwestern Spitsbergen, 77°00′N, 15°33′E). The analysis focuses on the influence of meteorological factors (snow depth, temperature), cosmic conditions, and solar activity on the radiation dose rate in the Arctic region over the period 2016–2024. The mean gamma dose rate during the investigated period was 0.0990 µSv/h (median: 0.1018 µSv/h), with daily average minimum and maximum values of 0.0676 µSv/h and 0.1324 µSv/h, respectively. In comparison to the global gamma average dose rate (0.0970 µSv/h), the observed levels do not pose an additional radiation exposure risk to individuals residing at the Polish Polar Station Hornsund. However, ongoing climate change, particularly the reduction in the duration and extent of snow cover, has led to an increase in the average gamma dose rate, which may have implications for populations inhabiting polar regions.

## Introduction

Natural gamma radiation measured at the Earth’s surface originates from radioactive isotopes present in the Earth’s crust and the interactions between cosmic radiation (CR) and the Earth’s atmosphere (Grieder 2001). Terrestrial radioactivity primarily results from radioactive nuclides in the Earth’s crust, particularly those belonging to the uranium (²³⁵U and ²³⁸U) and thorium (²³²Th) decay chains, as well as the long-lived radioactive isotope potassium (⁴⁰K). The external dose rate from gamma radiation is generally higher for the thorium decay chain than for the uranium chain (NAS 1999). Collectively, these sources contribute to the external radiation dose to which humans are exposed.

Furthermore, natural radioactivity is closely associated with radon (²²²Rn) emanation (Brajnik et al. 1992). Radon, a noble gas produced during the radioactive decay of ²³⁸U, represents a significant component of the total radiation dose. Due to its high mobility, radon readily escapes from the ground, making it the primary source of air ionization in the lower atmosphere (Pulinets 2007).

Although radon transport is significantly limited by the presence of permafrost during the freezing season, in summer — when the permafrost thaws to a depth of at least 3.0 m in the vicinity of the Polish Polar Station Hornsund (Majdański et al. 2022) — the conditions favour its release into the atmosphere (Marsz et al. 2013). Moreover, it plays a fundamental role in atmospheric electricity, exerting a considerable influence on the global electric circuit (Pulinets et al. 2024).

The relationship between solar activity and radiation dose rates has been studied in various environments, with a negative correlation between solar activity and ambient dose equivalent H*(10) observed on Earth (Zorko et al. 2021). In addition to solar influences, meteorological factors such as snow cover thickness, atmospheric pressure, temperature, precipitation, and substrate type and its structural properties also affect radiation dose rates (Ćujić et al. 2021). Moreover, snow cover plays a crucial role in attenuating ambient gamma dose rates and reducing terrestrial gamma-ray flux, as evidenced by multiple studies conducted in Japan (Omori et al. 2019).

Consequently, the degree of air ionization affects the quality of potential gradient and current density measurements (Harrison 2004; Tacza et al. 2020; Kubicki et al. 2021), which play a crucial role in the global electric circuit (GEC). Monitoring its behavior is essential due to its associations with various atmospheric phenomena, including lightning discharges (Nieckarz et al. 2009; Williams and Mareev 2014). Due to its unique geographical location in the polar region and its crucial importance for geophysical research, the radiation measurements and dose rate assessments conducted at the Polish Polar Station Hornsund are highly valuable, providing essential data for environmental monitoring and global atmospheric processes (Williams 2003).

This study includes an analysis of the gamma dose rate, considering both the influence of cosmic radiation modulated by solar activity and meteorological conditions. For the first time, we present the results from long-term measurements of radiation doses conducted at the Polish Polar Station Hornsund.

## Materials and methods

### Study area

**Fig. 1:**
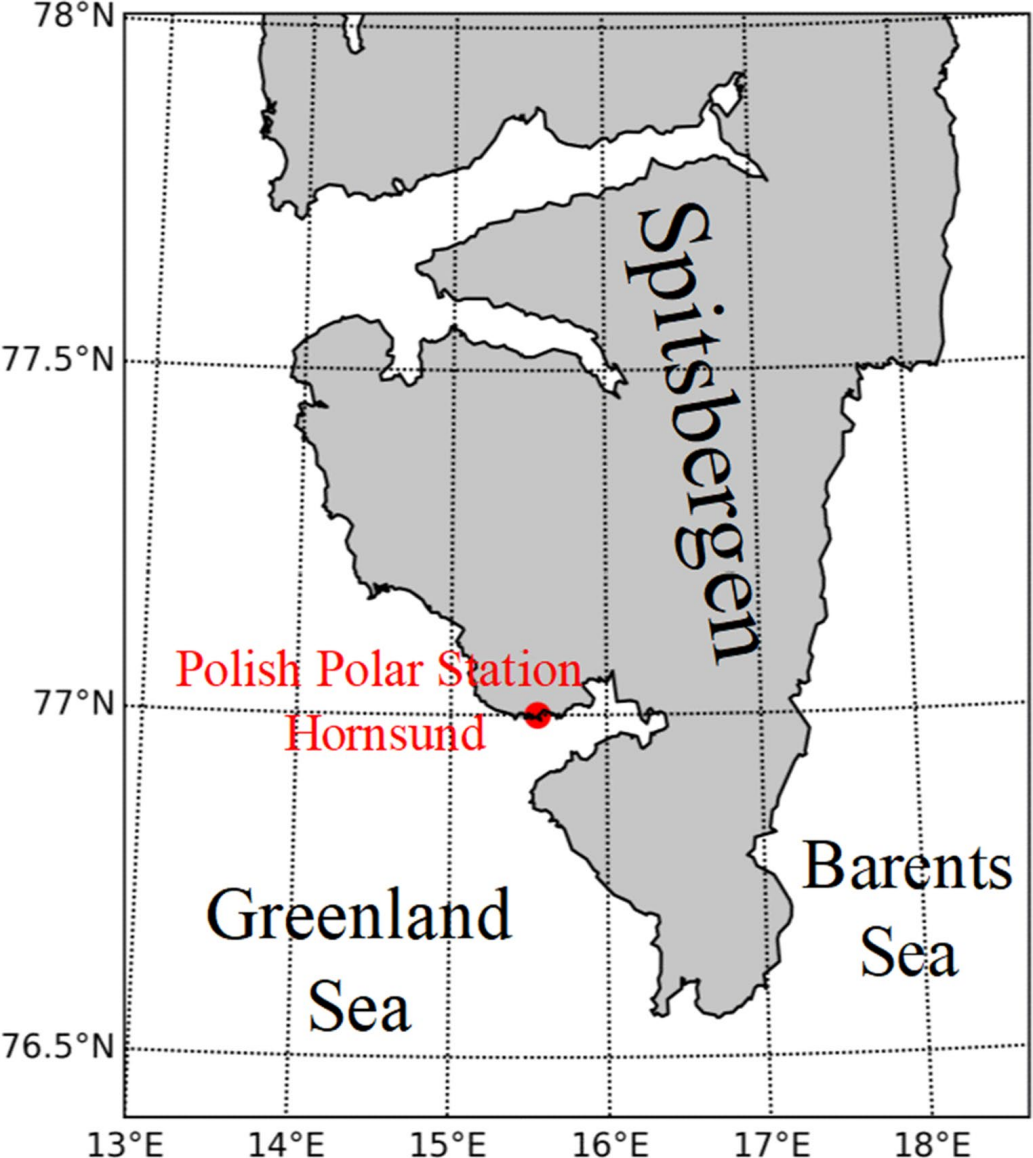
Map of the location of the Polish Polar Station Hornsund on Spitsbergen

This study presents the results of measurements conducted from October 4, 2016, to September 30, 2024, at the Stanisław Siedlecki Polish Polar Station Hornsund (77°00′N, 15°33′E), southwestern Spitsbergen, managed by the Institute of Geophysics, Polish Academy of Sciences. Established in 1957 during the International Geophysical Year, the station is situated on the northern shore of the Hornsund fjord, approximately 300 m from the coast of Isbjørnhamna Bay (Fig. 1). The station, located on a Holocene-raised marine terrace (10 m a.s.l.), is situated approximately 2.5 km from the Hansbreen glacier front, with the underlying permafrost extending to depths exceeding 100 m (Lindner et al. 1991; Wawrzyniak et al. 2016).

### Gamma dose rate measurements and instrumentation setup

Gamma dose rate measurements were conducted using the EcoGamma™-g (type Gamma Probe, model ECOGAMMA-g, SN 13001141, Canberra Industries Inc., USA) environmental gamma radiation monitor with thermal compensation (Canberra 2012). EcoGamma is a dual sensor consisting of a pair of Geiger-Müller counters as the basic detection elements. The instrument is a wide-range gamma dose rate detector (total range from 10 nSv/hr to 10 Sv/hr), compliant with H*10, and offering linearity within ± 10% referenced to Cesium-137 (Mirion 2012). The device can operate in two measurement ranges: a low range from 10 nSv/h to 5 mSv/h, and a high range from 0.05 mSv/h to 10 Sv/h. The data presented in this paper were acquired using the low dose rate range, which offers a sensitivity of 960 CPM/µSv/h. The instrument is capable of operating within a temperature range of −40 °C to + 60 °C and features automatic built-in calibration and linearity check functions, with test points and ranges that can be scaled to meet local regulatory requirements. Comprehensive calibration software is integrated into the unit, including full linearity verification capabilities.

The ambient dose equivalent H*(10) is recommended by the International Commission on Radiological Protection (ICRP 2007) as an operational quantity for assessing the effective dose in environmental monitoring, as it provides an easy and reliable estimation of the biological effects of radiation on humans. In the following sections of this study, the ambient dose equivalent H*(10) will be referred to simply as the dose rate.

**Fig. 2:**
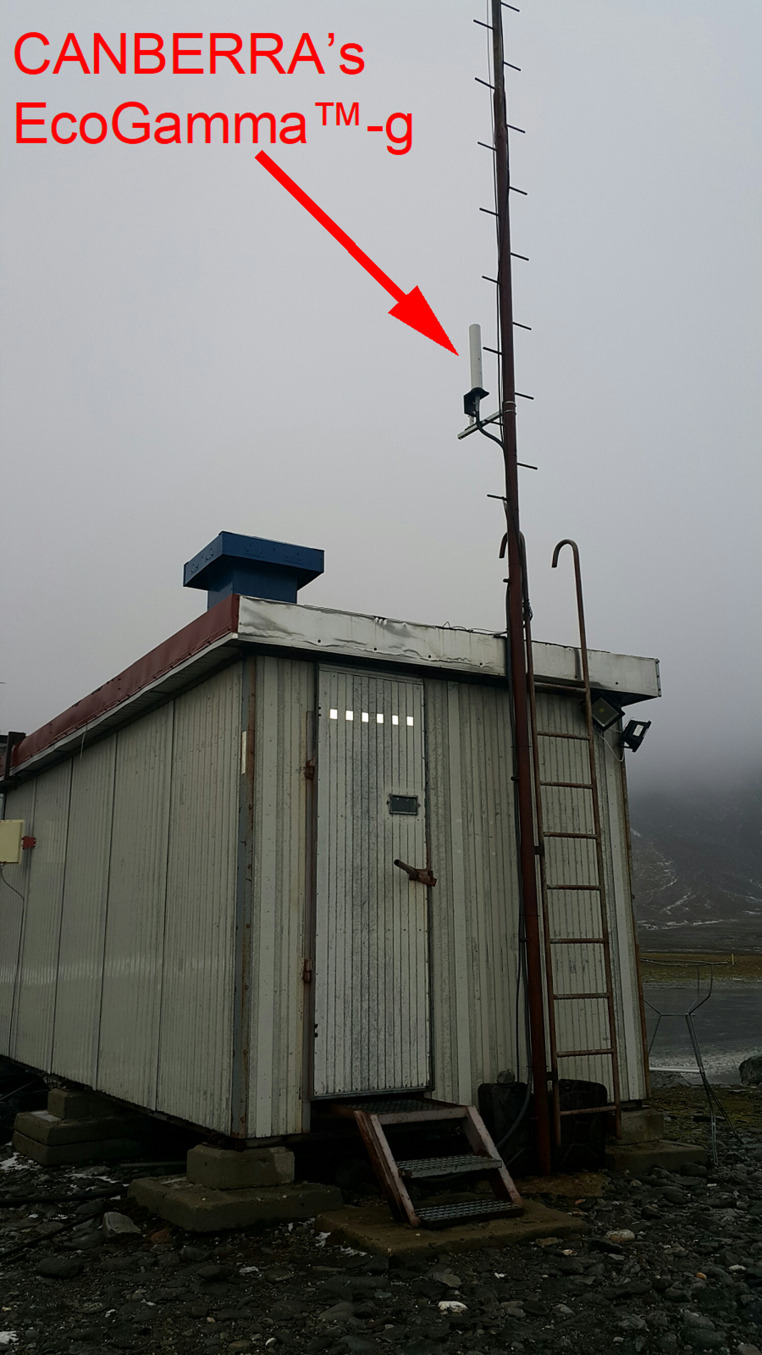
View of the container with CANBERRA EcoGamma™-g detector mounted on the mast

The device was mounted on a mast attached to the measurement container, positioned 5 m above ground level (Fig. 2). The detector was connected to a computer located inside the container, with dedicated software responsible for data acquisition and counter control. The container was situated 400 m northeast of the polar research base and 300 m from the coastline. The detector’s vertical orientation enables observations over a large solid angle. The detector is a component of the air radioactivity measurement system operating at the Polish Polar Station Hornsund since 2002 (Burakowska et al. 2021) and is the northernmost station (Latitude 77°).

Dose rate measurements, expressed in µSv/h, were recorded in files with a temporal resolution of one minute. Based on the collected data, hourly, daily, and monthly average dose rate values were calculated.

### Neutron dataset and detector specifications

Neutron count rate data were obtained from the Cosmic Ray Station (CRS) of the University of Oulu, which is part of the Sodankylä Geophysical Observatory (SGO) in Finland and has served as a key facility for cosmic ray research since 1964 (Usoskin et al. 2001). The data are available at the web page https://cosmicrays.oulu.fi. The CRS operates a 9-NM-64 neutron monitor (Niemi 1968), which is a standard type of ground-based cosmic ray detector. The system consists of the BF_3_-filled proportional gas counters arranged into three independent units (labeled A, B, and C), each comprising three counters. The Oulu neutron monitor is recognized as one of the most stable and reliable stations within the global Neutron Monitor Network (Usoskin et al. 1997). The effective vertical cutoff rigidity at the site is approximately 0.8 GV.

### Meteorological dataset

Comprehensive meteorological measurements are carried out in Hornsund using an automatic weather station and manually by an observer. The paper analyses selected meteorological parameters, i.e., air temperature and snow cover height. Air temperature has been measured at a height of 2 m using the Vaisala HMP 45D and HMP155 sensors (since January 2018). The measurement accuracy is 0.1 °C, and the time resolution is 10 s. Identical measurement conditions are used for measuring atmospheric pressure using the PTB 220 sensor. On this basis, the average daily and monthly air temperatures and pressure are calculated. The snow cover height is measured manually using the snow gauge every day at 06:00 UT. The accuracy of snow height measurement is ± 1 cm. The measured value represents the snow cover of the previous day.

A detailed description of all meteorological parameters measured at the Polish Polar Station Hornsund has been provided by Wawrzyniak and Osuch (2020).

### Assumptions underlying dose rate interpretation

In this study, the environmental gamma dose rate is considered to result from two primary sources: radiation originating from the Earth’s surface and cosmic radiation. We assume that during periods of frost—which limits radon diffusion from the ground, as discussed by Glover and Blouin (2022)—and when a thick snow cover is present, acting as a natural shield, the contribution of terrestrial radiation is significantly attenuated, as demonstrated by Omori et al. (2019). Under such conditions, the measured gamma dose rate is therefore attributed exclusively to cosmic radiation.

## Results and discussions

**Fig. 3:**
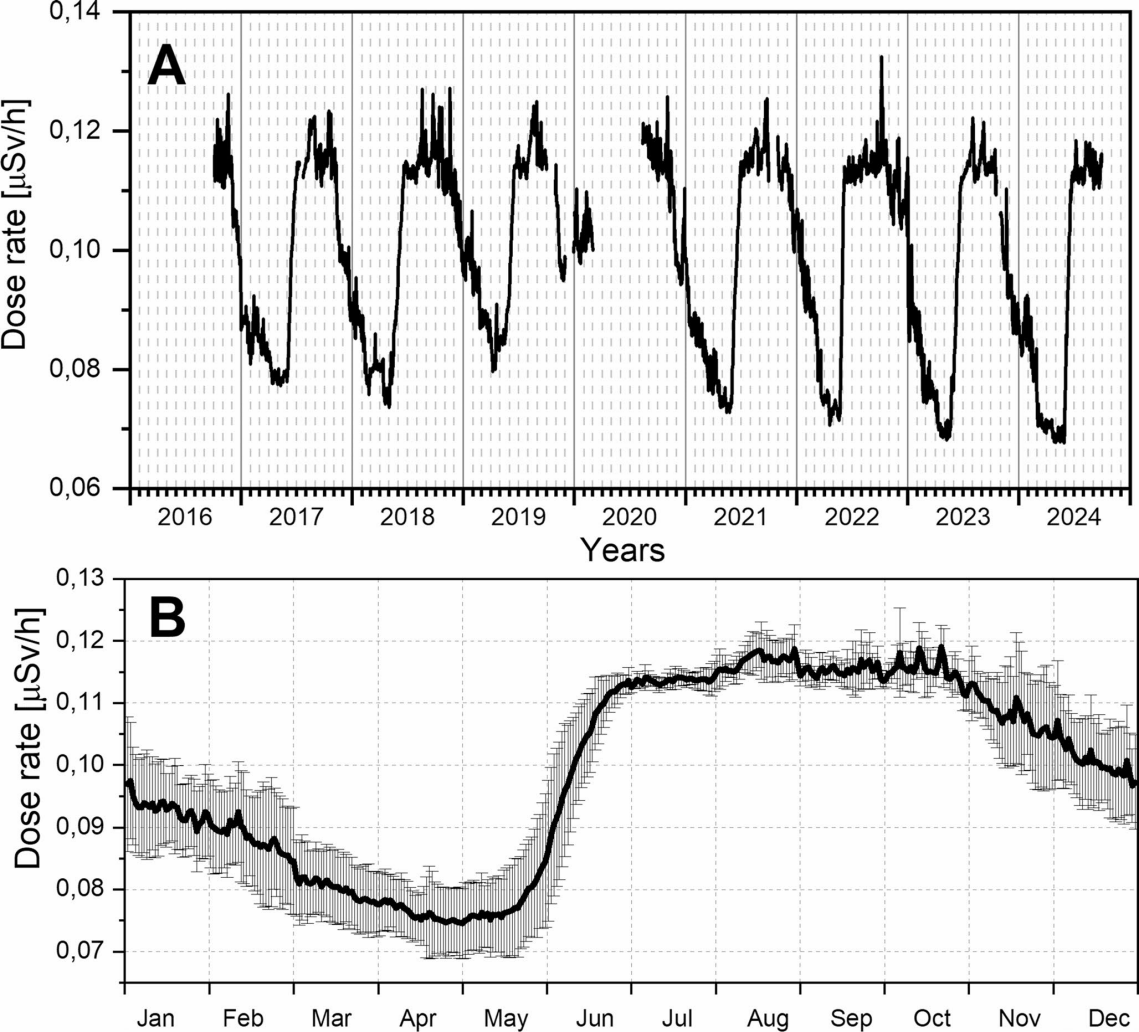
The plots shows the distribution of the mean daily gamma dose rate values (Panel A) and the average daily gamma dose rate profile with standard deviation range (Panel B)

The diurnal variation of gamma dose rate during the study period from October 4, 2016, to September 30, 2024, is presented in Fig. 3. The maximum dose rate of 0.13245 µSv/h was recorded on October 7, 2022, while the minimum dose rate of 0.06759 µSv/h was observed on May 29, 2024.

The mean gamma dose rate during the study period was 0.0990 µSv/h (median: 0.1018 µSv/h), which is comparable to average dose rate values measured across Europe (https://remap.jrc.ec.europa.eu/Advanced.aspx) (Rybach et al. 1997; Cinelli et al. 2018). The average daily gamma dose profile has the smallest standard deviation in July (see Fig. 3b).

**Fig. 4:**
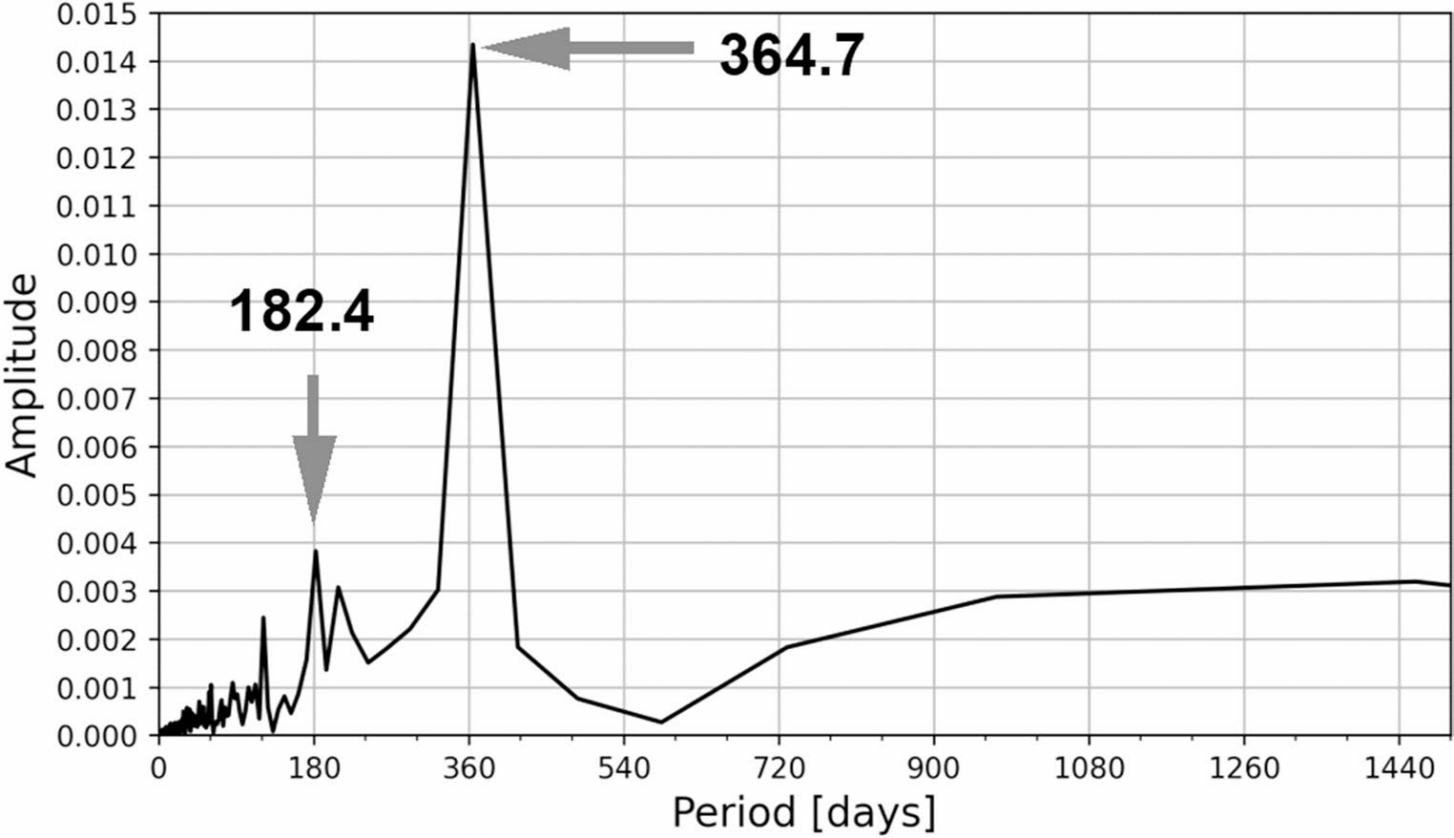
The amplitude spectrum calculated using the multitaper method based on the mean daily gamma dose rate

The amplitude periodogram, estimated using the multitaper method (Slepian 1978; Thompson 1982) and based on mean daily dose rate values, is presented in Fig. 4. The analysis reveals the two most prominent periods — 364.7 and 182.4 days — corresponding to the annual and semi-annual cycles, respectively. The amplitude of the annual component is approximately 3.7 times greater than that of the semi-annual one.

**Fig. 5:**
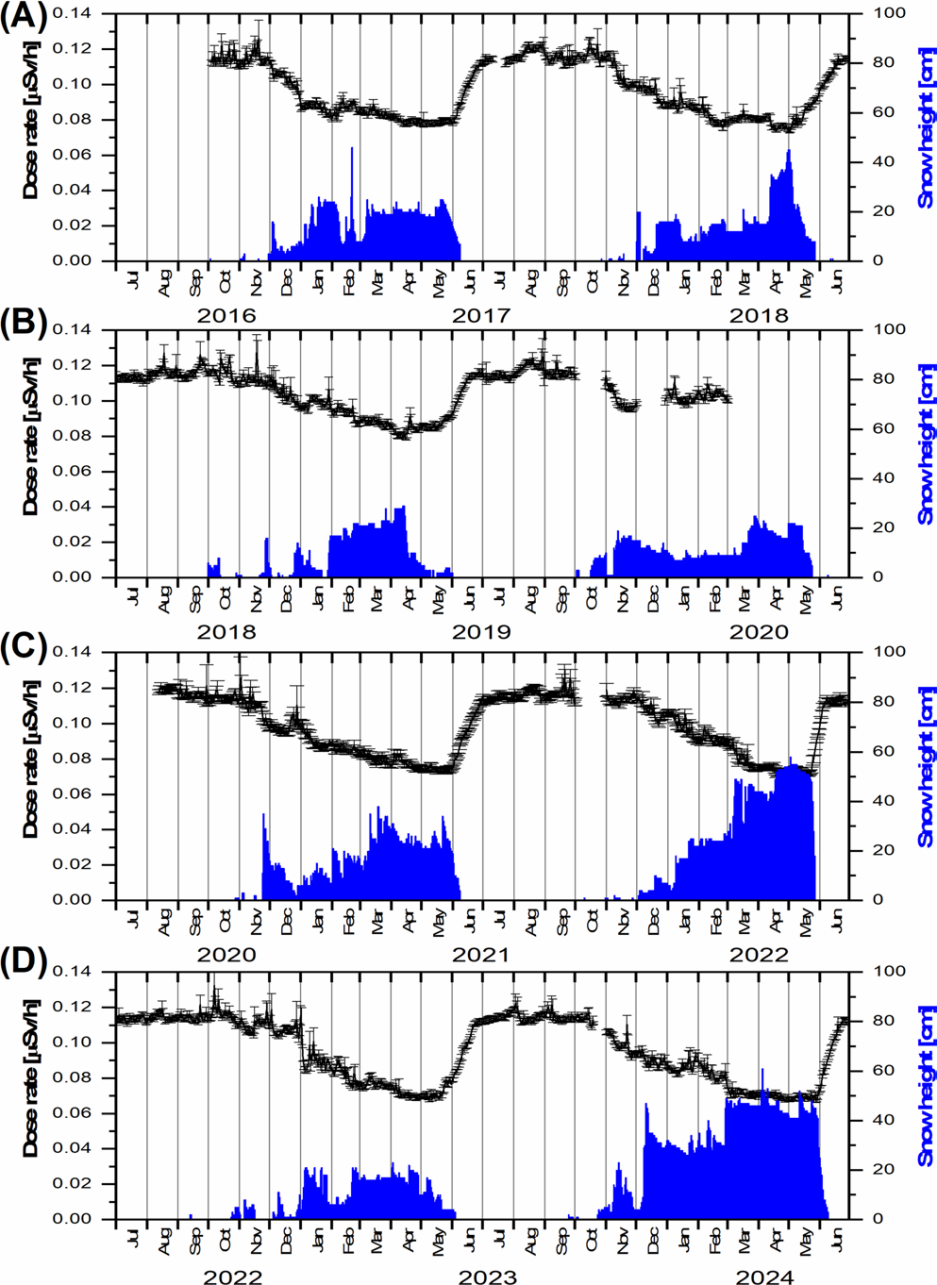
The variation of mean daily gamma dose rate with standard deviation and snow height values recorded at the Polish Polar Station Hornsund; daily snow height measurements are performed with an accuracy of ± 1 cm (not shown in the figure)

Daily average radiation gamma dose rates and snow cover depth over the study period from October 4, 2016, to September 30, 2024, are depicted in Fig. 5. The highest recorded snow cover depth, measuring 61 cm, was observed on April 5, 2024.

**Fig. 6:**
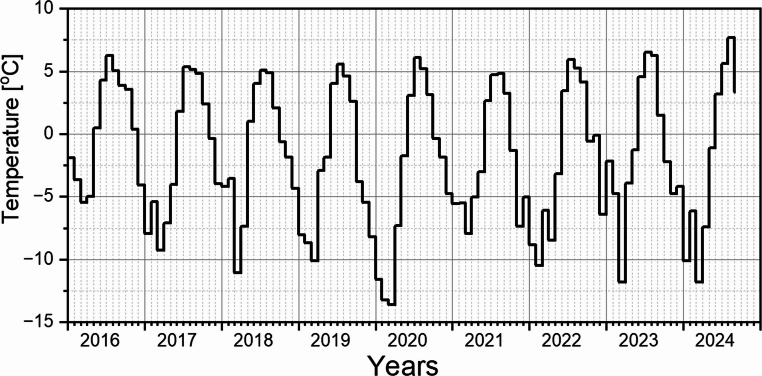
The course of the monthly air temperature recorded at the Polish Polar Station Hornsund

The recurring sharp increase in radiation gamma dose rate values observed at the end of May (or early June, depending on the year) correlates with the rapid disappearance of the snow cover (see Fig. 5). Snowmelt is a natural consequence of rising air temperatures (Fig. 6) and the associated transfer of thermal energy.

**Fig. 7:**
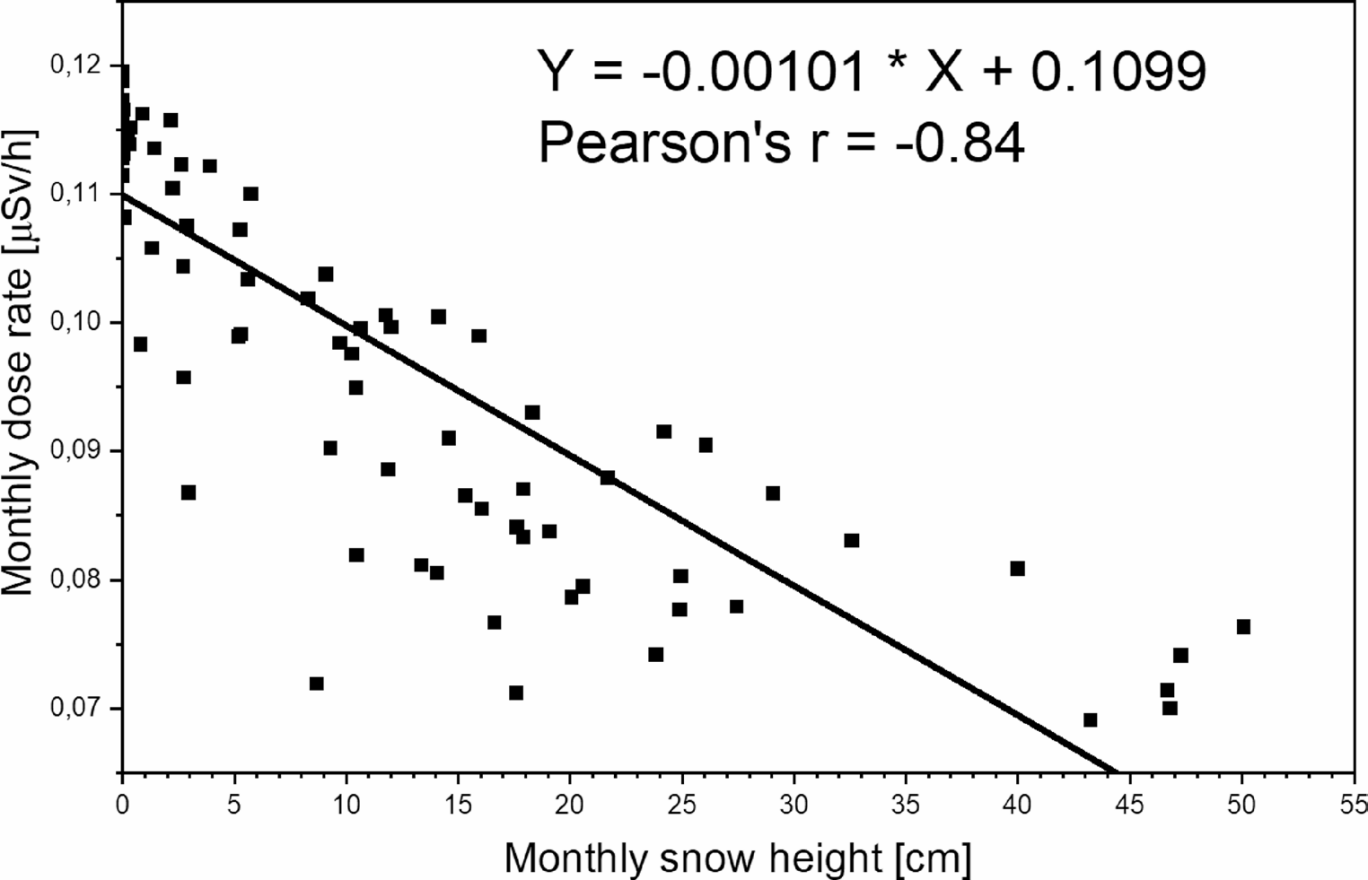
The scatterplot illustrating the relationship between the monthly dose rate and monthly snow height, along with its corresponding linear fit

The relationship between monthly radiation gamma dose values and monthly snow cover depth is presented in Fig. 7. The results of the linear fit indicate a statistically significant association between the two variables, with a slope of −0.00101 at a significance level of *p* < 0.001 and a correlation coefficient of −0.84. This value indicates a strong negative relationship between the two variables. This finding is consistent with studies conducted in Japan (Omori et al. 2019). Snow cover acts as a shielding layer, attenuating gamma radiation emitted from the ground. A higher correlation coefficient could likely have been obtained if the calculations had accounted for temporal variations in the average density of the snowpack. Unfortunately, no such measurements were carried out.

**Table 1:** The minimum daily gamma dose rate values and average gamma dose rate, average snow height, monthly SSN, and neutron counts rate (and their standard deviations, ±SD) in April recorded during the years 2017–2024 (where * means data with gaps)

Years	2017	2018	2019	2020	2021	2022	2023	2024
Minimum daily dose rate value (± SD) [µSv/h]	0.0772 (± 0.0011)	0.0736 (± 0.0011)	0.0796 (± 0.0011)	0.0942* (± 0.0015)	0.0728 (± 0.0013)	0.0706 (± 0.0013)	0.0681 (± 0.0015)	0.0676 (± 0.0010)
Average dose rate (± SD) [µSv/h] in April	0.0795 (± 0.0015)	0.0779 (± 0.0025)	0.0833 (± 0.0027)	-	0.0777 (± 0.0028)	0.0741 (± 0.0017)	0.0712 (± 0.0022)	0.0700 (± 0.0011)
Average snow height (± SD) [cm] in April	20.6 (± 1.2)	27.4 (± 10.2)	17.9 (± 9.2)	18.5 (± 2.2)	24.9 (± 2.8)	47.3 (± 5.1)	17.6 (± 2.9)	46.8 (± 3.9)
Monthly SSN (± SD) in April	32.3 (± 24.6)	8.9 (± 8.7)	9.1 (± 7.6)	5.2 (± 8.2)	24.5 (± 19.9)	84.0 (± 39.4)	97.6 (± 40.0)	136.5 (± 76.5)
Neutron counts rate (± SD) [cts/min] in Oulu for April	6616 (± 81)	6678 (± 82)	6732 (± 82)	6813 (± 82)	6737 (± 82)	6576 (± 81)	6135 (± 78)	5914 (± 77)

The minimum daily gamma dose rate occurs during the winter season. Interannual variations in its values exhibit a similar pattern to the mean gamma dose values recorded in April across different years (Table 1). For both parameters under consideration, elevated values were observed in 2017–2018, followed by a monotonic decline during 2021–2024. This trend cannot be solely explained by snow cover thickness, as in the years 2021–2024, the April snow cover depth exhibited significant fluctuations, alternating between values below 25 cm (in 2021 and 2023) and above 45 cm (in 2022 and 2024), without a clear correlation with the observed radiation dose values (Table 1). This suggests that beyond a certain minimum snow cover thickness, cosmic factors become the dominant influence.

In addition to blocking radiation from the ground, snow cover also reduces the emission of radon isotopes (²²⁰Rn and ²²²Rn), which constitute the primary source of air ionisation in the atmospheric boundary layer (Pulinets 2007). Radon isotopes emanate from the ground into the atmosphere, decaying into various progeny, some of which act as gamma radiation emitters (e.g., ²¹⁴Bi, ²¹⁴Pb, and ²⁰⁸Tl), thereby increasing the radiation dose (Casanovas et al. 2016).

An additional factor limiting radon release from the soil is the presence of sub-zero temperatures, which cause ground freezing and hinder gas exchange between the soil and the atmosphere, consequently restricting radon migration into the air. The solubility of radon in water is directly proportional to its partial pressure above the water surface and depends on water temperature, increasing at lower temperatures. Furthermore, radon solubility is influenced by the water’s pH and mineralisation. These factors collectively contribute to an increase in radon concentration in the air with rising temperatures (Schubert et al. 2012).

**Fig. 8:**
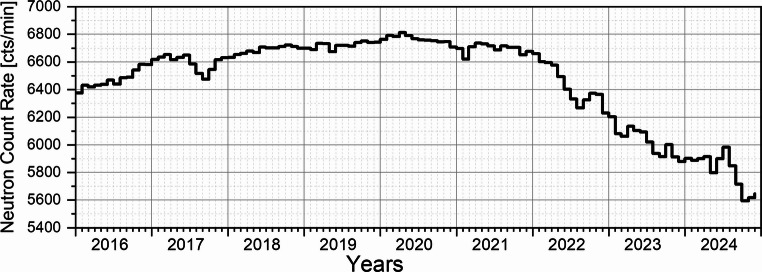
Monthly average distribution of corrected neutron count rates registered at the Oulu Neutron Monitor. (source: https://cosmicrays.oulu.fi)

It should be noted that the contribution of radiation from the Earth’s crust to the total dose rate decreases under conditions of sub-zero temperatures and snow cover presence. In such circumstances, the minimum radiation dose is primarily determined by the intensity of cosmic radiation. Solar activity modulates the cosmic radiation flux (the so-called Forbush effect; Belov 2008), whereby an increase in solar activity leads to a reduction in the cosmic radiation reaching the Earth’s surface. This effect is confirmed by measurements of secondary neutrons (Usoskin et al. 2001), which originate from the interactions of cosmic radiation particles with atmospheric molecules (the so-called particle cascades) and are recorded by the Neutron Monitor in Oulu (Fig. 8).

**Fig. 9:**
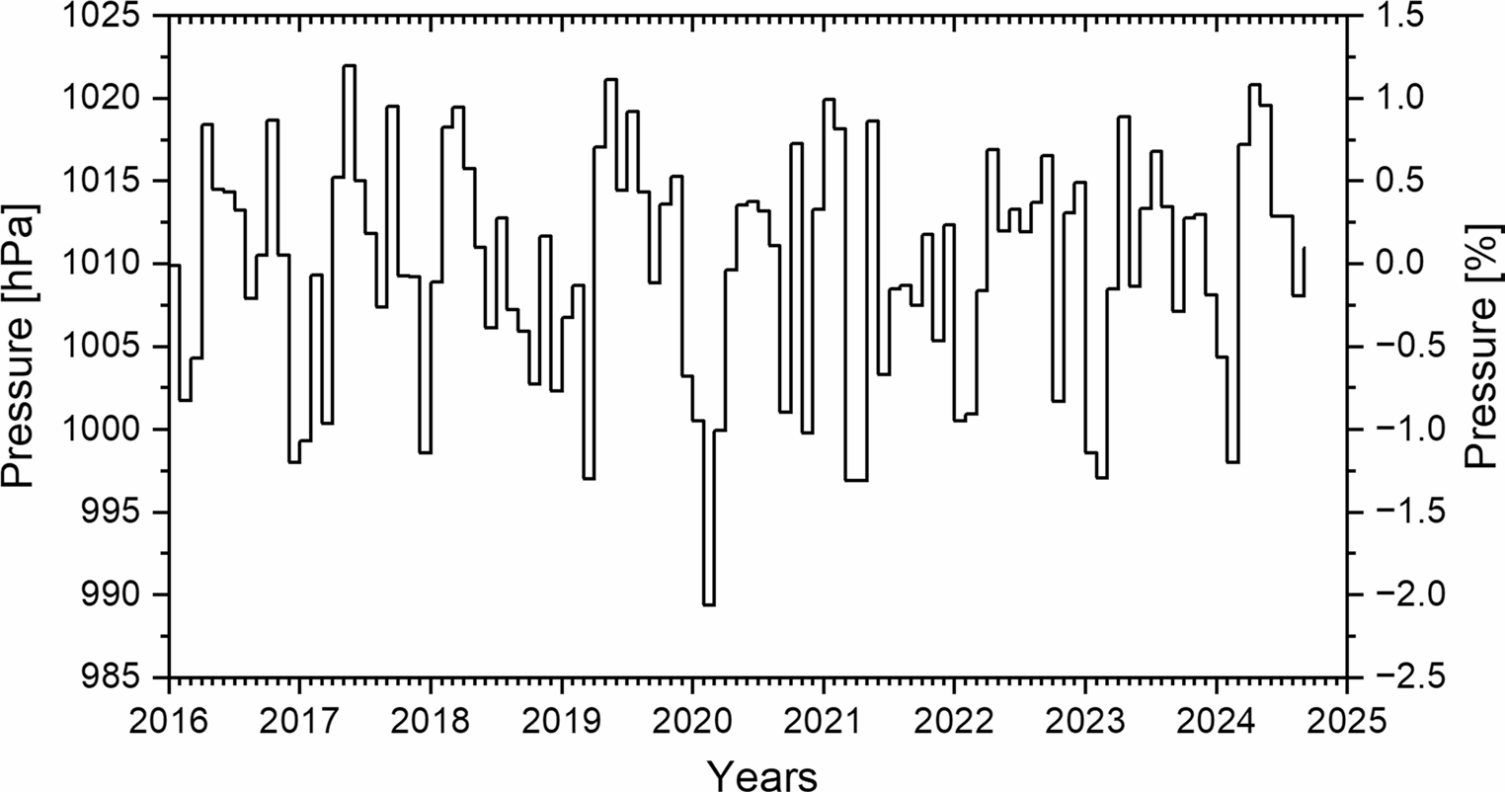
The course of the monthly air pressure recorded at the Polish Polar Station Hornsund

It should also be noted that both neutron count rates and dose rates exhibit a few per cent variability due to fluctuations in atmospheric pressure (Wissmann 2005) and seasonal variations (Jeong and Oh 2022). The pressure variations recorded at the Polish Polar Station Hornsund during the study period remain within ± 1.5% of the mean value (Fig. 9). These fluctuations are minor in comparison to the over 70% increase in gamma dose rate observed between the winter period (minimum values of approximately 0.07 µSv/h) and the summer period (maximum values of approximately 0.12 µSv/h).

**Fig. 10:**
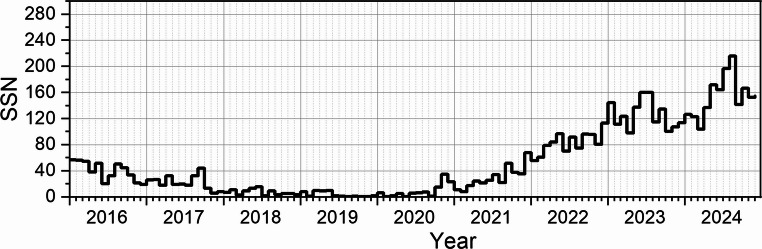
Monthly total sunspot number. (source: https://www.sidc.be/SILSO/datafiles)

**Fig. 11:**
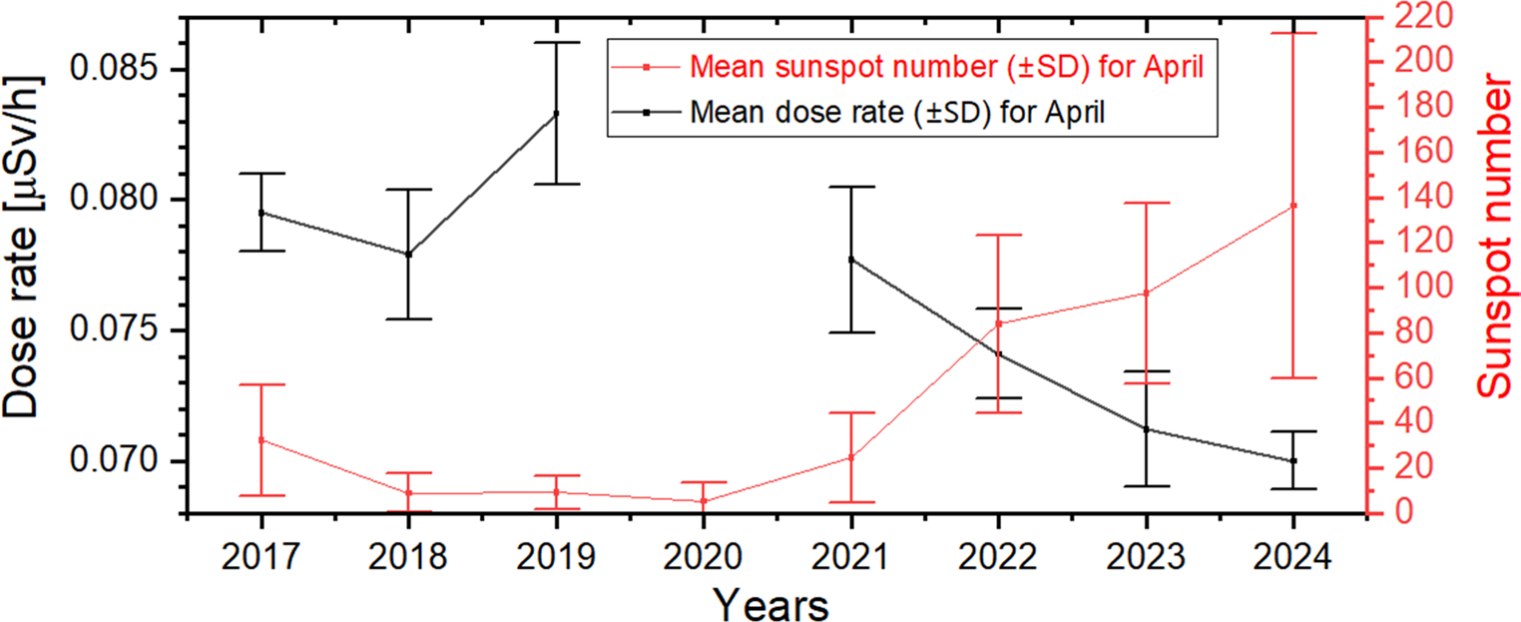
Distribution of the dose rate (from gamma radiation) and the average sunspot number (SSN) observed in April from 2017 to 2024, both shown with standard deviation ranges

The period from 2016 to 2020 corresponds to the passive phase of low solar activity during Solar Cycle 24 (Fig. 10), whereas the years 2021–2024 fall within the active phase of Solar Cycle 25 (Zięba and Nieckarz 2014). Figure 11 demonstrates that during the solar minimum, the monthly dose rate from gamma radiation recorded at the Polish Polar Station Hornsund for April in the years 2017–2019 was approximately 14% higher compared to the solar maximum period (2023–2024).

Furthermore, cosmic radiation also contributes indirectly to a slight increase in the dose rate through spallation reactions involving oxygen and nitrogen nuclei in the upper atmosphere, leading to the production of *⁷Be*. This isotope, through deep vertical mixing processes within the atmosphere, is transported towards the Earth’s surface (Burakowska et al. 2021).

## Conclusions

During the study period, the mean gamma dose rate was 0.099 µSv/h, exceeding both the global average of 0.097 µSv/h (Thorne 2003; UNSCEAR 2008) and the value measured in Poland in 2023, which was 0.075 µSv/h (PAA 2023). It is important to emphasize that the recorded radiation level is not considered high, as higher gamma dose rate values have been observed in numerous regions, including Norway (Komperød et al. 2015). Thus, staying at the Polish Polar Station Hornsund does not significantly increase human radiation exposure.

When compared to southern polar regions, the recorded values fall within the range of gamma dose rates (0.042–0.380 µSv/h) observed at various Antarctic research stations (Nakajima et al. 1995; Kruetzmann 2006; Guillaume and Sébastien 2017). Notably, in certain locations, such as the Indian research station Bharati, significantly higher dose rates have been reported (up to 0.7 µSv/h; Bakshi et al. 2013).

It is worth highlighting that extremely low gamma dose rate values are only observed during the winter season, when thick snow cover is present, and during the active phase of solar activity.

Ongoing climate change is leading to an increase in air temperature and, consequently, a reduction in the depth of the ground freezing zone. According to reports (Osuch and Wawrzyniak 2017; Kavan and Haagmans 2021), a decreasing trend in the maximum snow cover thickness has been observed on Spitsbergen, with a decline of −5.7 cm per decade, alongside a reduction in the duration of snow cover by 10.0 days per decade in the vicinity of the Polish Polar Station Hornsund. As a result, ionizing radiation emitted from the ground is no longer attenuated as effectively, and radon is more readily released from the soil, both of which contribute to an increase in gamma dose rates around the station. A similar trend has been reported by Glover and Blouin (2022), who highlight that subarctic populations may be exposed to hazardous radon levels. For this reason, it is crucial to continue and expand gamma dose rate monitoring in polar regions.

Further variations in dose rates will affect the degree of air ionisation, the distribution of the electric field, and the density of currents recorded at the Earth’s surface (Pulinets et al. 2024). Consequently, future research and the development of models for the Earth’s global electric circuit (Rycroft and Harrison 2011) will need to account for changes in radiation dose within the atmospheric boundary layer.

## Data Availability

Data will be available on request.
